# Processes Underpinning Development and Maintenance of Diversity in Rice in West Africa: Evidence from Combining Morphological and Molecular Markers

**DOI:** 10.1371/journal.pone.0085953

**Published:** 2014-01-20

**Authors:** Alfred Mokuwa, Edwin Nuijten, Florent Okry, Béla Teeken, Harro Maat, Paul Richards, Paul C. Struik

**Affiliations:** 1 Knowledge, Technology and Innovation Group (KTI), Wageningen University, Wageningen, the Netherlands; 2 Louis Bolk Institute, Driebergen, the Netherlands; 3 Africa Rice Center, Cotonou, Bénin; 4 School of Environmental Sciences, Njala University, Njala, Sierra Leone; 5 Centre for Crop Systems Analysis, Wageningen University, Wageningen, the Netherlands; University of Nottingham, United Kingdom

## Abstract

We assessed the interplay of artificial and natural selection in rice adaptation in low-input farming systems in West Africa. Using 20 morphological traits and 176 molecular markers, 182 farmer varieties of rice (*Oryza* spp.) from 6 West African countries were characterized. Principal component analysis showed that the four botanical groups (*Oryza sativa* ssp. *indica*, *O. sativa* ssp. *japonica*, *O. glaberrima*, and interspecific farmer hybrids) exhibited different patterns of morphological diversity. Regarding *O. glaberrima,* morphological and molecular data were in greater conformity than for the other botanical groups. A clear difference in morphological features was observed between *O. glaberrima* rices from the Togo hills and those from the Upper Guinea Coast, and among *O. glaberrima* rices from the Upper Guinea Coast. For the other three groups such clear patterns were not observed. We argue that this is because genetic diversity is shaped by different environmental and socio-cultural selection pressures. For *O. glaberrima*, recent socio-cultural selection pressures seemed to restrict genetic diversity while this was not observed for the other botanical groups. We also show that *O. glaberrima* still plays an important role in the selection practices of farmers and resulting variety development pathways. This is particularly apparent in the case of interspecific farmer hybrids where a relationship was found between pericarp colour, panicle attitude and genetic diversity. Farmer varieties are the product of long and complex trajectories of selection governed by local human agency. In effect, rice varieties have emerged that are adapted to West African farming conditions through genotype × environment × society interactions. The diversity farmers maintain in their rice varieties is understood to be part of a risk-spreading strategy that also facilitates successful and often serendipitous variety innovations. We advocate, therefore, that farmers and farmer varieties should be more effectively involved in crop development.

## Introduction

West African farmers have cultivated two species of rice *Oryza sativa* (Asian rice) and *Oryza glaberrima* (African rice) for several centuries. Over much of the West African coastal zone, resource-poor farmers cultivate the two species as rainfed varieties in a range of ecologies, from lowland to upland. According to one view, Asian rice was introduced into coastal West Africa by Portuguese traders in the 16th century [1]. Another view is that it may have arrived earlier (perhaps around the beginning of the Common Era) via trans-Saharan trade routes and trade links between East Africa and India [2]. African rice (*O*. *glaberrima*) is thought to have been first domesticated in the swampy basins of the upper Niger River delta 3000–4000 years ago [3], [4]. Since its introduction into West Africa, Asian rice has tended to replace African rice, particularly in wetland cultivation. From the late 18th century onwards a second wave of introductions occurred from Asia and America, including both *O. sativa* ssp. *indica* and *O. sativa* ssp. *japonica*. This boosted the rate at which *O. sativa* replaced *O. glaberrima* [3], now including in dryland rice farming conditions. This accelerated replacement, alongside the enduring cultivation of *O. glaberrima* in certain pockets, is often explained as resulting from local variations in socio-cultural, political, ecological and geographical factors influencing farmers and their work [5]–[9]. *O. glaberrima* is widely believed to be well adapted to low-input farming conditions [10].

*Oryza glaberrima* has never been improved by agronomists or plant breeders. Professional opinion has been that the species has little to offer and that yields are invariably low. More recently, *O*. *glaberrima* has been seen as a useful genetic resource to improve *O. sativa* varieties [11], [12]. The two rice species are genetically isolated from each other by an F1 sterility barrier ([13]–[17], amongst others), although gene exchange can occur in the field [15], [17]–[21]. Recent research confirms that varieties with an interspecific background, resulting from introgressions, are regularly to be found in farmer fields along the Upper Guinea Coast from The Gambia down to Sierra Leone [22], [23]. Because backcrossing to either parent (to produces fertile progeny) results in parental phenotypical resemblance, it is difficult to detect hybrid derivatives; they look like either *sativa* or *glaberrima* [17], [23]. This means that four botanical clusters can be identified as co-existing in West Africa: these are *O. sativa* ssp. *indica*, *O. sativa* ssp. *japonica*, *O. glaberrima* and interspecific farmer varieties [23].

A recurrent idea in the literature is that although farmer varieties look very diverse morphologically, they are actually genetically rather uniform at gene pool level because of continuous selection on qualitative traits in the same gene pool [24] and because most farmer varieties are the result of recombination of existing farmer varieties [25]. A common, different view is that farmer varieties are made up of different genotypes, making them genetically quite diverse. Both views do not seem to apply to rice in West Africa. The first idea is countered by a study conducted by Nuijten et al. [23], and the second view may apply to other crops, but not to rice [6]. In West Africa the coexistence of Asian and African rice has resulted in an enlarged gene pool and the development of interspecific farmer varieties [23], [26], [27]. The main underlying factors are farmer selection and gene flow through cross pollination and seed exchange [6]. From seemingly isolated hamlets seed can travel long distances, through informal seeds networks, mostly based on extended family ties, and can diffuse across countries [7], [28]. These processes of seed diffusion have been traced over several centuries [29]. The time-depth and durability of this process prepares us to understand the finding that farmer varieties can embody greater levels of genetic diversity than formal varieties [30], challenging an assumption often made by plant breeders that the reverse is true on account of the access enjoyed by breeders to a world-wide spectrum of genetic resources [31]. The existence of farmer varieties with an interspecific background clearly shows that farmer crop development has more potential value as a complement to scientific breeding than is often assumed [23]. The value of these activities, by farmers in West African conditions, is further reinforced by recent research showing that farmer rice varieties can be adapted to a wide range of agro-ecological conditions [10].

Country-specific studies have been conducted to unravel the genetic variability of rice in West Africa (e.g. for Sierra Leone, Guinea and The Gambia, see [22], [30], [32], [33]). Nuijten et al. [23] then offered a regional perspective by analysing a large set of farmer varieties collected from seven countries across coastal West Africa, using molecular markers. To obtain a more complete understanding of the processes underlying the development and maintenance of genetic diversity, the present study now combines molecular and morphological characterization with socio-economic information concerning four botanical groups of rice from six West African countries. The aim is to explain how farmer practices have combined with environmental pressures to shape rice diversity in the case study countries. Reference to historical and socio-cultural data is made in order to better understand region-specific morphological traits.

**Figure 1:**
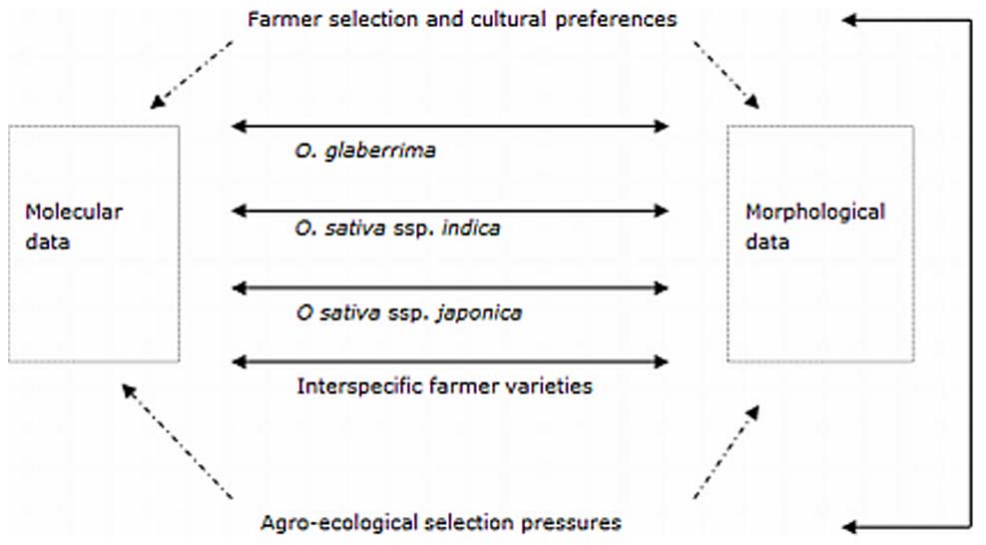
Schematic representation of the main aspects of our research and their interlinkages.

Analysis directs attention to underlying processes regulating the development of genetic diversity in crops in low-input farming systems - processes not yet well understood. An important issue is to grasp the scope of the interplay of artificial and natural selection in crop adaptation. Our findings suggest (Figure 1) that there are multiple pathways for natural and artificial (farmer) selection to influence molecular and morphological markers. Correlations between morphological and molecular data may also vary among the botanical groups because of differences in genetic background, robustness and differential response to human or environmental selection pressures.

Our analysis confirms that rice varieties in West Africa are adapted to their conditions as a result of genotype × environment × society interactions. The rice diversity farmers appeared to maintain is probably part of a risk-spreading strategy that facilitates innovations in variety development.

## Results and Discussion

### Rice Diversity in West Africa at the Molecular Level

**Figure 2:**
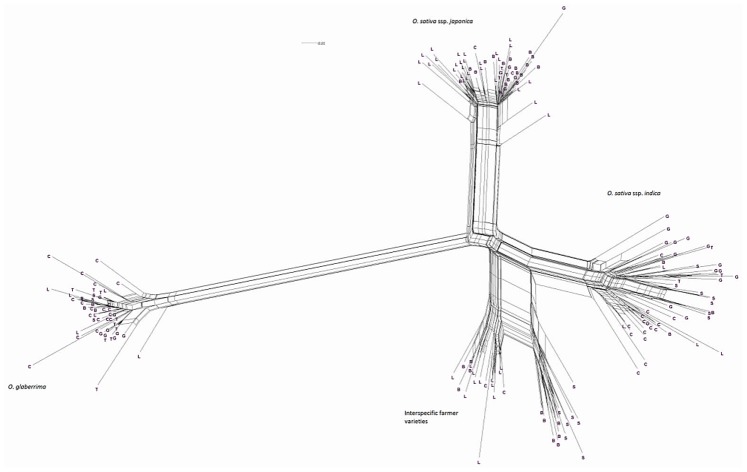
Phylogenetic relationships based on molecular markers of the 182 materials included in the morphological analysis. Country of collection is indicated by letters: B  =  Guinea **B**issau, C  =  Guinea **C**onakry, G  =  **G**hana, L  =  Sierra **L**eone, S  =  **S**enegal, T  =  **T**ogo.

Figure 2 illustrates the phylogenetic relationships of materials studied in the field trial, as assessed during molecular analysis (cf. [23]). Four clusters are shown in detail. Three of these clusters correspond to the botanical groups *O. glaberrima*, *O. sativa* ssp. *japonica* and *O. sativa* ssp. *indica*. In between *O. glaberrima* and *O. sativa* ssp. *indica* is situated the group of interspecific farmer varieties sharing the genetic background of both *O. glaberrima* and *O. sativa* (see [23], hereafter referred to as Cluster 4).

The genotypes comprising each cluster also tend to separate in sub-clusters (Figure 2). The genotypes of the *O. glaberrima* cluster split into *O. glaberrima* from the lower Guinea Coast and *O. glaberrima* from the upper Guinea Coast. The *indica* group splits into several sub-clusters in a complex way. Some sub-clusters only consist of genotypes from one country (*indica* from Ghana), while other sub-clusters are constituted by materials from different countries. The *japonica* cluster splits into one sub-cluster with *japonica* mainly from Sierra Leone and a sub-cluster with *japonica* mainly from Ghana and Guinea Bissau. The cluster of the farmer hybrids splits into one sub-cluster with genotypes that display erect and semi-erect panicles and a second sub-cluster with droopy panicles. Genotypes of the first sub-cluster (Cluster 4-1) were found in Sierra Leone, Guinea and Guinea Bissau while genotypes of the second sub-cluster were found in Guinea Bissau and Senegal (Cluster 4-2). The following sections explore the morphological diversity of the respective sub-clusters to see how they are related to the observations at molecular level and farming system level. Various historical and contextual explanations for these clusterings are discussed in Nuijten et al. [23] and Mouser et al. [29]. For example, the Ghana-Guinea Bissau *japonica* cluster could be interpreted as indicating a pathway of rice introduction from the East Indies via the important and long-established Portuguese coastal trading stations at Elmina (Ghana) and Cacheu (Guinea Bissau).

### Morphological Diversity

**Figure 3:**
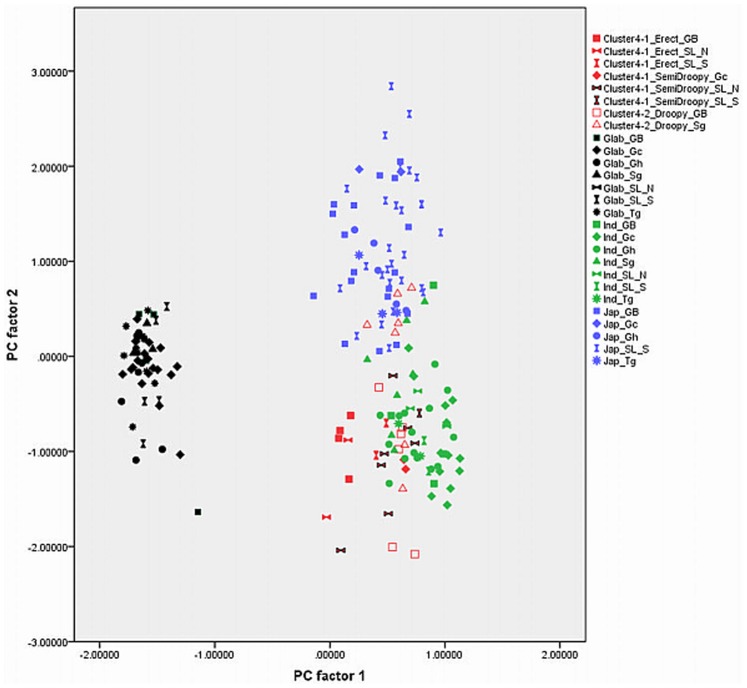
Graphical repartition of materials based on morphological data of PC 1 and 2. **Component 1**: Ligule shape (0.97)*, Leaf blade pubescence (0.90), Ligule length (0.90), PAMA** (0.88), PAB*** (0.77), Rattoon potential (0.74), Leaf blade colour (0.65). **Component 2**: Leaf width (0.80), # tillers per plant (−0.79), # panicles per plant (−0.79), Seed width (0.71), Leaf blade colour (0.50). Glab: *glaberrima*, Ind: *indica*, Jap: *japonica*, Clusters 4-1 and 4-2: farmer hybrids. GB: Guinea Bissau, SL: Sierra Leone (north: N south: S), Gc: Guinea Conakry, Sg: Senegal, Gh: Ghana, Tg: Togo. *(): value of the correlation of the trait with the component. **: Panicle Attitude of Main Axis. ***: Panicle Attitude of Branches.

**Figure 4:**
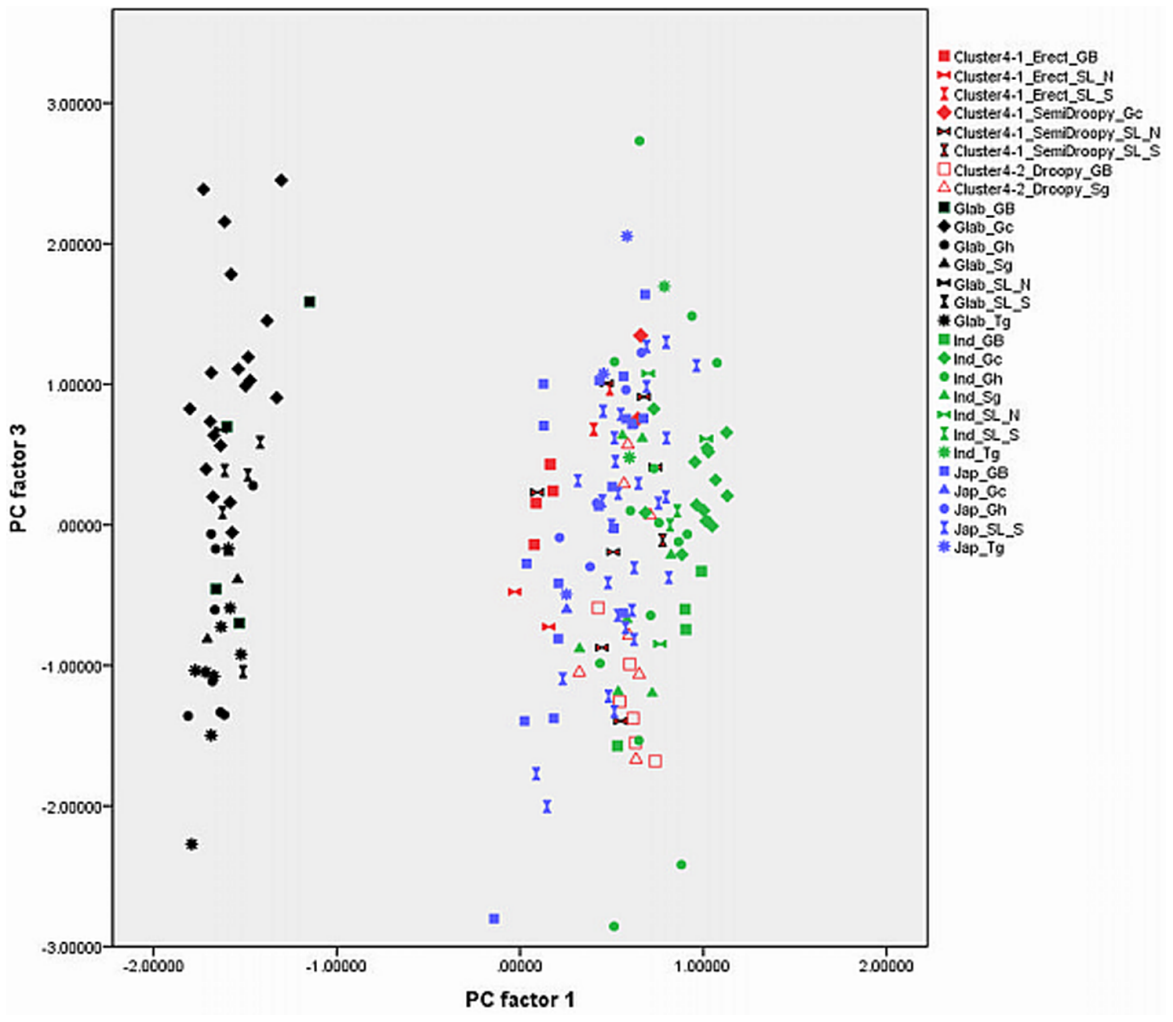
Graphical repartition of materials based on morphological data of PC 1 and 3. **Component 1**: Ligule Shape (0.97)*, Leaf blade pubescence (0.90), Ligule length (0.90), PAMA** (0.88), PAB*** (0.77), Rattoon potential (0.74), Leaf blade colour (0.65). **Component 3**: Plant height (0.95), Culm length (0.88), Panicle length (0.70), Leaf length (0.60). GB: Guinea Bissau, SL: Sierra Leone (north: N south: S), Gc: Guinea Conakry, Sg: Senegal, Gh: Ghana, Tg: Togo. *(): value of the correlation of the trait with the component. **: Panicle Attitude of Main Axis. ***: Panicle Attitude of Branches.

**Figure 5:**
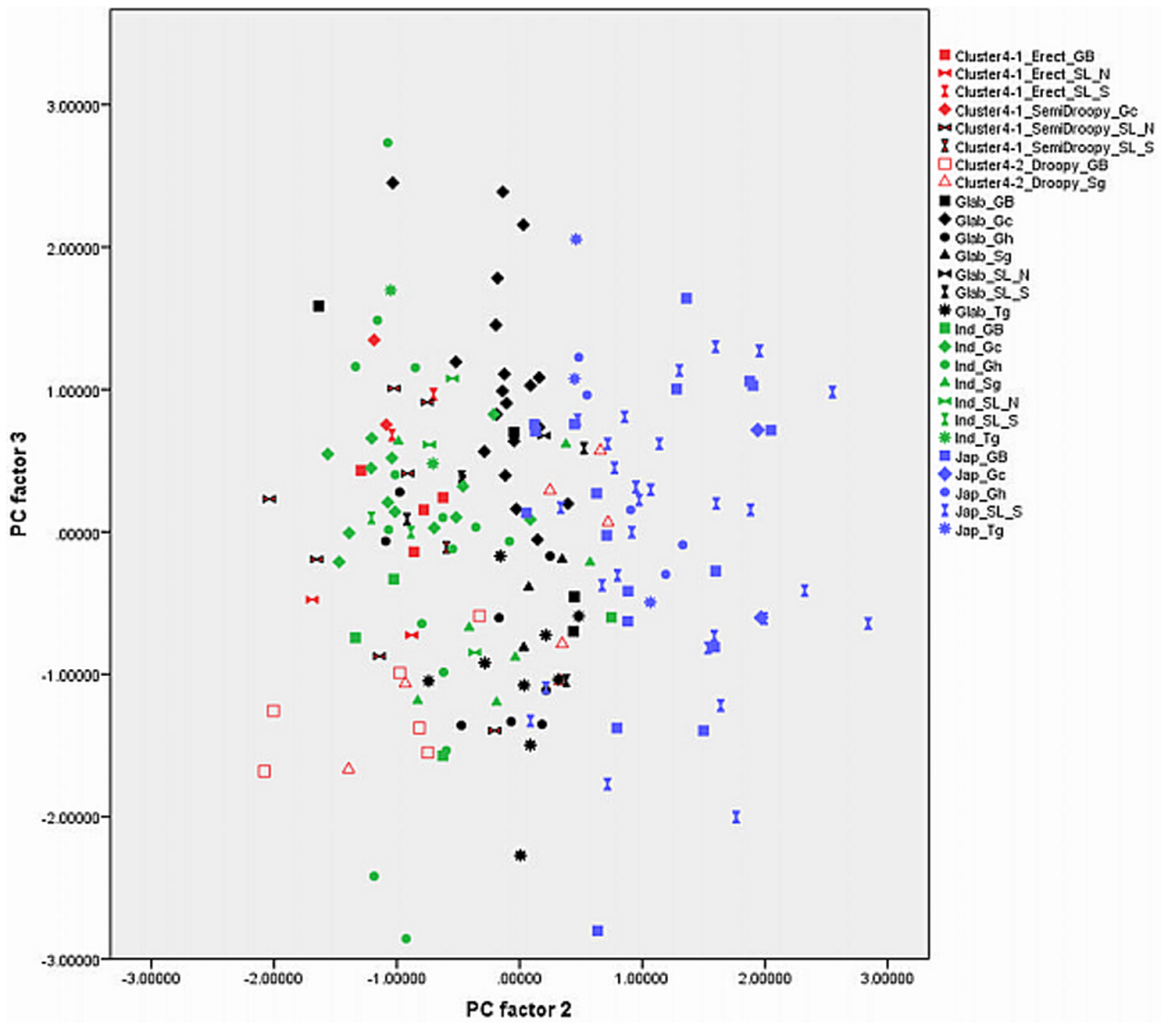
Graphically repartition of materials based on morphological data of PC 2 and 3. **Component 2**: Leaf width (0.80)*, # tillers / plant (−0.79), # panicles/plant (−0.79), Seed width (0.71), Leaf blade colour (0.50). **Component 3**: Plant height (0.95), Culm length (0.88), Panicle length (0.70), Leaf length (0.60). GB: Guinea Bissau, SL: Sierra Leone (north: N south: S), Gc: Guinea Conakry, Sg: Senegal, Gh: Ghana, Tg: Togo. *(): value of the correlation of the trait with the component.

**Figure 6:**
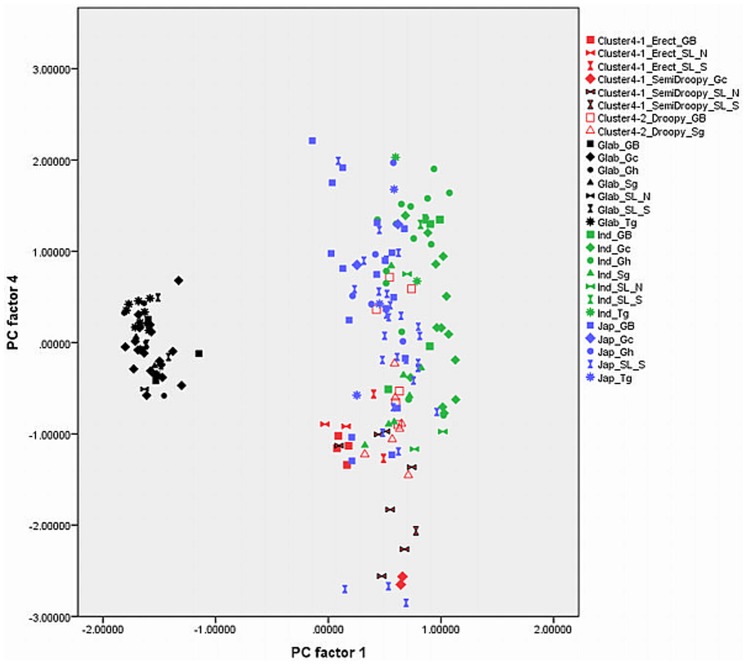
Graphically repartition of materials based on morphological data of PC 2 and 3. **Component 1**: Ligule Shape (0.97)*, Leaf blade pubescence (0.90), Ligule length (0.90), PAMA** (0.88), PAB*** (0.77), Rattoon potential (0.74), Leaf blade colour (0.65). **Component 4**: Seed length (0.93), Seed width (−0.36). GB: Guinea Bissau, SL: Sierra Leone (north: N south: S), Gc: Guinea Conakry, Sg: Senegal, Gh: Ghana, Tg: Togo. *(): value of the correlation of the trait with the component. **: Panicle Attitude of Main Axis. ***: Panicle Attitude of Branches.

**Figure 7:**
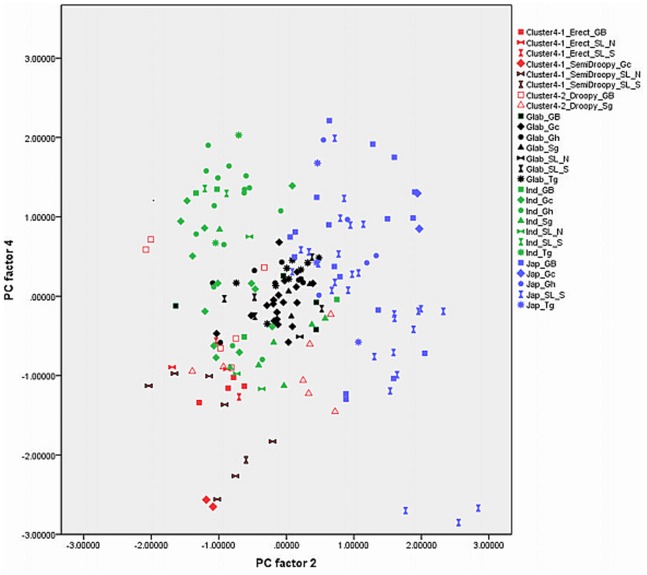
Graphically repartition of materials based on morphological data of PC 2 and 4. **Component 2**: Leaf width (0.80)*, # tillers / plant(−0.79), # panicles/plant (−0.79), Seed width (0.71), Leaf Blade Colour (0.50). **Component 4**: Seed length (0.93), Seed width (−0.36). GB: Guinea Bissau, SL: Sierra Leone (north:N south: S), Gc: Guinea Conakry, Sg: Senegal, Gh: Ghana, Tg: Togo. *(): value of the correlation of the trait with the component.

**Figure 8:**
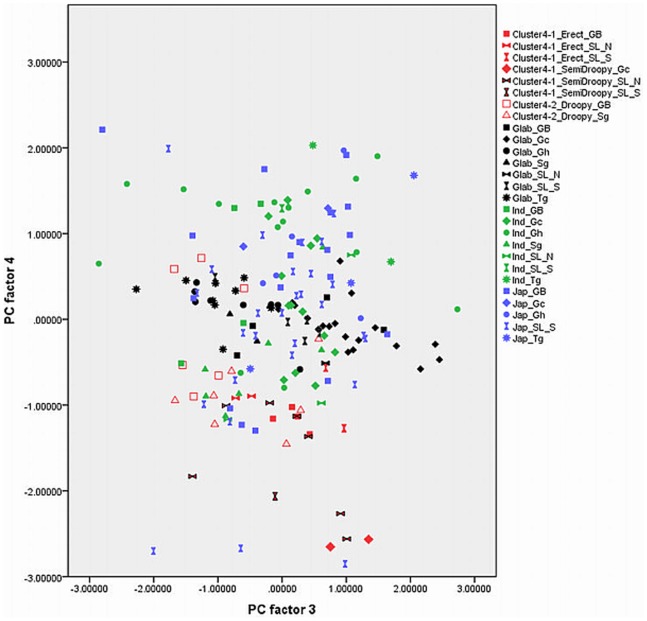
Graphically repartition of materials based on morphological data of PC 3 and 4. **Component 3**: Plant height (0.95)*, Culm length (0.88), Panicle length (0.70), Leaf length (0.60). **Component 4**: Seed length (0.93), Seed width (−0.36). GB: Guinea Bissau, SL: Sierra Leone (north: N, south: S), Gc: Guinea Conakry, Sg: Senegal, Gh: Ghana, Tg: Togo. *(): value of the correlation of the trait with the component.

Out of 17 principal components (PCs), the first four accounted for 73.57% of the variance among the traits studied (Table 1). The fifth component was not used in the biplots (Figures 3, 4, 5, 6, 7, and 8) because it had very little explanatory value for most traits. Table 2 presents the rotated principal components matrix and shows how traits contributed to the PCs. Traits commonly used to distinguish *O. glaberrima* from *O. sativa* contributed most to PC 1: ligule shape and length, panicle attitude of main axis (PAMA), and leaf blade pubescence. Traits that contributed most to PC 2 were leaf width, seed width, number of tillers and number of panicles. Traits that contributed most to PC 3 were culm length, plant height, panicle length and leaf length. Seed length contributed clearly to PC 4. Tables 3 and 4 show average values, standard deviations and coefficients of variation for 10 agronomic traits, by botanical groups and sub-groups.

**Table 1:** Initial eigenvalues and rotation sums of squared loadings of 17 principal components based on 17 morphological traits measured on 182 rice accessions.

Principal Component	Initial eigenvalues			Rotation sums of squared loadings		
	Total	% of variance	Cumulative %	Total	% of variance	Cumulative %
1	5.98	35.17	35.17	5.51	32.38	32.38
2	3.13	18.38	53.55	2.98	17.51	49.90
3	2.20	12.94	66.49	2.65	15.57	65.47
4	1.20	7.08	73.57	1.29	7.58	73.04
5	1.00	5.90	79.47	1.09	6.43	79.47
6	0.88	5.16	84.63			
7	0.64	3.79	88.42			
8	0.58	3.42	91.84			
9	0.36	2.09	93.93			
10	0.29	1.70	95.63			
11	0.23	1.38	97.01			
12	0.21	1.22	98.23			
13	0.12	0.71	98.94			
14	0.08	0.47	99.41			
15	0.06	0.34	99.76			
16	0.04	0.24	100.00			
17	0.00	0.00	100.00			

Extraction Method: Principal Component Analysis.

**Table 2:** Rotated Principal Components (PCs) of 17 morphological rice traits.

Trait	Components				
	1	2	3	4	5
Leaf blade colour	0.65	0.50	−0.04	−0.10	−0.05
Leaf blade pubescence	0.90	0.02	0.01	−0.16	0.07
Culm length	−0.09	0.05	0.88	−0.15	0.24
Plant height	−0.09	0.02	0.95	−0.10	0.12
Panicle length	−0.04	−0.09	0.70	0.17	−0.40
Leaf length	0.25	0.37	0.60	0.14	0.20
Leaf width	−0.37	0.80	0.25	0.05	0.12
Ligule length	0.90	−0.22	0.05	−0.02	−0.01
Ligule shape	0.97	0.07	−0.07	−0.00	0.02
# tillers / plant	−0.42	−0.79	−0.05	−0.07	0.11
# panicles / plant	−0.44	−0.79	−0.00	−0.08	0.07
Panicle attitude of mainaxis (PAMA)	0.88	0.16	−0.09	0.28	−0.07
Panicle attitude ofbranches (PAB)	0.77	0.28	−0.07	0.17	−0.04
Seed length	0.10	−0.07	−0.05	0.93	0.09
Seed width	−0.05	0.71	−0.05	−0.36	0.19
Collar colour	0.05	0.03	0.17	0.09	0.81
Rattoon potential	0.74	0.15	0.08	0.23	0.27

Extraction Method: Principal Component Analysis; Rotation Method: Varimax with Kaiser Normalization.

**Table 3:** Means, standard deviation (SD), coefficient of variation (CV) and t-test results (P, in bold) for the agronomic morphological traits for the different botanical groups from the Lower Guinea Coast (Lower) and the Upper Guinea Coast (Upper).

Botanical group		*Glaberrima*		*Indica*		*Japonica*		Average
Region		Lower	Upper	Lower	Upper	Lower	Upper	all groups
Trait	N	17	32	29	17	8	48	151
Culm length (cm)	Mean	79.1	86.3	81.0	82.5	89.2	82.4	83.4
	SD	5.10	6.52	8.37	6.25	5.09	5.72	6.18
	CV (%)	6.45	7.55	10.33	7.57	5.71	6.94	7.43
	**P**		**0.000**		**0.528**		**0.003**	
Plant height (cm)	Mean	99.1	109.7	103.3	104.7	110.2	103.9	105.2
	SD	5.22	7.09	9.21	6.17	6.27	6.52	6.75
	CV (%)	5.27	6.46	8.91	5.89	5.68	6.27	6.41
	**P**		**0.000**		**0.561**		**0.014**	
Panicle length (cm)	Mean	20.1	23.4	22.3	22.2	21.1	21.5	21.8
	SD	1.01	1.29	1.44	1.01	1.95	2.23	1.49
	CV (%)	5.03	5.50	6.46	4.55	9.24	10.37	6.86
	**P**		**0.000**		**0.800**		**0.611**	
Leaf length (cm)	Mean	41.4	42.3	43.1	42.8	48.7	46.3	44.1
	SD	2.80	2.52	4.17	2.65	3.22	3.91	3.21
	CV (%)	6.77	5.96	9.66	6.19	6.62	8.45	7.28
	**P**		**0.234**		**0.761**		**0.107**	
Leaf width	Mean	1.44	1.46	1.04	1.06	1.50	1.58	1.34
	SD	0.07	0.12	0.10	0.14	0.08	0.20	0.12
	CV (%)	5.04	8.14	9.62	13.17	5.62	12.57	9.03
	**P**		**0.140**		**0.572**		**0.261**	
# tillers / plant	Mean	5.2	4.8	4.3	4.6	3.3	3.1	4.2
	SD	0.80	0.80	0.58	0.64	0.46	0.59	0.65
	CV (%)	15.48	16.46	13.32	14.05	14.11	19.12	15.42
	**P**		**0.599**		**0.208**		**0.485**	
# panicles / plant	Mean	4.5	4.4	3.8	4.0	2.7	2.7	3.7
	SD	0.56	0.70	0.52	0.60	0.30	0.47	0.53
	CV (%)	12.51	16.19	13.98	15.05	10.89	17.39	14.34
	**P**		**0.621**		**0.123**		**0.738**	
Average 200 seed weight (g)	Mean	4.63	4.17	4.67	4.77	5.29	4.82	4.73
	SD	0.19	0.41	0.38	0.44	0.33	0.73	0.41
	CV (%)	4.10	9.83	8.07	9.12	6.29	15.08	8.75
	**P**		**0.000**		**0.421**		**0.082**	
Seed length (mm)	Mean	8.69	8.40	9.13	9.03	9.25	8.66	8.86
	SD	0.16	0.29	0.68	0.60	0.49	0.82	0.51
	CV (%)	1.84	3.45	7.47	6.68	5.28	9.51	5.71
	**P**		**0.001**		**0.629**		**0.058**	
Seed width (mm)	Mean	3.07	3.03	2.93	2.96	3.18	3.25	3.07
	SD	0.07	0.13	0.30	0.19	0.24	0.31	0.21
	CV (%)	2.28	4.29	10.27	6.26	7.50	9.52	6.69
	**P**		**0.112**		**0.651**		**0.545**	
Average CV (%)		5.9	7.6	8.9	8.0	7.0	10.5	

**Table 4:** Means, standard deviations (SD), coefficients of variation (CV) and t-test results (P, in bold) for the agronomic morphological traits for the four different sub-groups within the Upper Guinea Coast.

Botanical group		*Glaberrima*		*Indica*		*Japonica*		Cluster 4		Cluster 4-1	
Region / sub-cluster		UpperGc	Upper-Other	UpperGc	Upper-Other	UpperGB	UpperSL	Cluster4-2	Cluster4-1	4-1Erect	4-1 Semi-droopy
Trait	N	19	13	13	12	18	28	13	18	8	10
Culm length (cm)	Mean	89.3	82.1	84.9	82.6	83.9	81.6	76.3	83.3	83.6	83.0
	SD	5.66	5.32	4.76	5.39	6.83	4.98	5.23	5.32	4.13	6.33
	CV (%)	6.34	6.48	5.61	6.53	8.14	6.11	6.86	6.39	4.94	7.63
	**P**		**0.001**		**0.260**		**0.198**		**0.001**		**0.826**
Plant height (cm)	Mean	112.8	105.2	107.1	104.3	105.1	103.2	97.9	104.6	104.3	104.9
	SD	6.25	5.80	4.70	5.84	7.95	5.68	5.76	6.04	4.51	7.28
	CV (%)	5.54	5.52	4.39	5.60	7.57	5.50	5.89	5.77	4.32	6.94
	**P**		**0.001**		**0.192**		**0.349**		**0.004**		**0.834**
Paniclelength (cm)	Mean	23.6	23.3	22.2	21.7	21.1	21.6	21.6	21.4	20.7	22.0
	SD	1.08	1.57	1.05	1.17	2.06	2.23	0.98	1.43	1.13	1.44
	CV (%)	4.59	6.75	4.76	5.38	9.75	10.32	4.55	6.68	5.47	6.57
	**P**		**0.543**		**0.352**		**0.479**		**0.642**		**0.060**
Leaf length (cm)	Mean	43.1	41.1	43.6	41.5	46.1	46.3	38.5	45.6	48.2	43.6
	SD	1.77	3.01	2.04	2.82	4.05	4.03	4.21	3.45	3.34	1.84
	CV (%)	4.10	7.32	4.68	6.79	8.77	8.70	10.93	7.55	6.94	4.22
	**P**		**0.043**		**0.041**		**0.892**		**0.000**		**0.002**
Leaf width	Mean	1.49	1.40	1.08	1.05	1.51	1.62	1.04	1.01	1.05	0.98
	SD	0.13	0.07	0.15	0.14	0.23	0.18	0.18	0.09	0.09	0.09
	CV (%)	8.92	4.72	13.99	13.76	15.08	10.85	17.11	9.22	8.26	9.13
	**P**		**0.030**		**0.633**		**0.087**		**0.672**		**0.104**
# tillers/plant	Mean	4.7	5.0	4.7	4.3	3.2	3.1	4.2	4.7	4.9	4.6
	SD	0.66	0.97	0.63	0.66	0.61	0.56	1.02	0.63	0.47	0.74
	CV (%)	13.85	19.51	13.62	15.31	19.32	18.05	24.44	13.45	9.73	16.14
	**P**		**0.367**		**0.240**		**0.874**		**0.101**		**0.391**
# panicles/plant	Mean	4.3	4.4	4.1	3.8	2.7	2.7	3.6	4.2	4.3	4.1
	SD	0.43	1.00	0.57	0.54	0.48	0.44	0.92	0.72	0.57	0.84
	CV (%)	9.91	22.79	13.90	14.38	17.83	16.42	25.84	17.44	13.32	20.83
	**P**		**0.808**		**0.128**		**0.819**		**0.049**		**0.529**
Average 200 seed weight (g)	Mean	4.04	4.35	4.73	4.93	5.03	4.58	3.73	3.59	4.37	2.96
	SD	0.20	0.54	0.49	0.21	0.67	0.61	0.61	0.79	0.30	0.39
	CV (%)	4.95	12.41	10.29	4.28	13.36	13.31	16.27	22.16	6.81	13.14
	**P**		**0.064**		**0.196**		**0.024**		**0.580**		**0.000**
Seed length (mm)	Mean	8.37	8.45	8.98	8.78	8.95	8.43	8.13	7.51	8.20	6.96
	SD	0.32	0.25	0.54	0.66	0.72	0.84	0.51	0.82	0.25	0.68
	CV (%)	3.82	2.96	6.00	7.51	8.01	9.92	6.28	10.90	3.05	9.78
	**P**		**0.469**		**0.425**		**0.035**		**0.015**		**0.000**
Seed width (mm)	Mean	2.96	3.12	2.96	3.15	3.20	3.27	2.84	2.90	3.09	2.74
	SD	0.08	0.14	0.15	0.28	0.31	0.32	0.40	0.21	0.04	0.16
	CV (%)	2.70	4.49	4.92	9.01	9.69	9.71	14.07	7.36	1.26	5.98
	**P**		**0.002**		**0.062**		**0.469**		**0.632**		**0.000**
Average CV (%)		5.9	8.5	7.5	8.0	10.7	9.9	12.0	9.7	5.8	9.1

UpperSL  =  material from Sierra Leone; UpperGB  =  material from Guinea Bissau; UpperGc  =  material from Guinea; Cluster 4-1  =  material belonging to the sub-cluster of Cluster 4 with erect and semi-droopy panicles; Cluster 4-2  =  material belonging to the sub-cluster of Cluster 4 with droopy panicles; 4-1 Erect  =  material of Cluster 4-1 with erect panicle; 4-1 semi droopy  =  material belonging to Cluster 4-1 with semi-droopy panicle.

#### Comparison between botanical groups

Figures 3, 4, 5, 6, 7, and 8 represent the morphological diversity using different combinations of PC 1, 2, 3 and 4. The graphical representation of genotypes using PC 1 and 2 (53.6%) shows two clouds of genotypes (Figure 3), separating *glaberrima* distinctly from the other three groups. *O*. *glaberrima* has a rounded and short ligule, erect panicle, erect primary branches, generally displays little leaf blade pubescence and tends to have a rather light leaf blade colour. This separation agrees with separations achieved through the molecular analysis.

By contrast, the three other botanical groups are not as clearly separated as they are in the molecular analysis. The clusters *japonica*, and *indica* form two connected clouds distributed along PC 2. The *japonicas* produce fewer tillers and panicles, and wider leaves and seed compared to the *indicas*. The farmer hybrids overlap mostly with the *indicas*. The molecular analysis also suggested that farmer hybrids are more closely related to *indicas* than to *japonicas* (see Figure 2). Most of the farmer hybrids that are clearly separate from the *indicas* belong to Sub-cluster 4-1 (Sierra Leone and Guinea Bissau) and only a few to Sub-cluster 4-2 (Guinea Bissau).

The combination of PC 1 and 3 (Figure 4) shows a larger overlap between *japonicas*, *indicas* and the farmer hybrids along the third component while the *glaberrima* cluster is pulled apart along the third component. The genotypes of *glaberrima* studied here are thus highly differentiated from each other on traits represented by PC 3 (plant height, culm length, panicle length and leaf length). The genotypes from Togo and Ghana tend to sit toward the lower part of the cloud and those from Guinea and Sierra Leone sit in the upper part.

When combining PC 2 and 3 (31.3%) all botanical groups form a single cloud (Figure 5). Whereas PC 1 is based on traits that separate *glaberrima* from the other botanical groups, PC 2 and 3 are based on a majority of the agronomic traits included in this study. The *glaberrimas*, *indicas* and most of the farmer hybrids, except for most of those from Senegal, are situated towards the left of the scatter, while the *japonicas* are positioned towards the right. Also, Figure 5 shows that *glaberrima* varieties differ more in height-related traits and panicle length than in number of panicles and tillers and leaf and seed width.

Through combination of PC 1 and 4 (Figure 6) the *glaberrima* group is bunched into a concentrated cluster showing a large degree of uniformity in related traits. The *indica* and *japonica* genotypes, however, show an equally large range for seed length. The farmer hybrids (mostly the erect and semi-droopy types) are situated at the lower part of the shared cloud with *indicas* and *japonicas,* showing relatively short grain length.

#### Comparison within botanical groups

When combining PC 1 and 3 (Figure 4) the *O. glaberrima* varieties from the Upper Guinea Coast are found in the upper part of the cloud, with those from Guinea right at the top, and those from the Lower Guinea Coast further down. The *glaberrimas* from the Upper Guinea Coast are taller and have longer culms and panicles but have similar leaf length and ligule length, when compared to the *glaberrimas* from the Lower Guinea Coast (Table 3). Among the Upper Guinea Coast *glaberrimas*, the varieties from Guinea seem to constitute a special group, being taller, with longer culms, panicles and leaves (Table 4). This was also observed in several trials conducted in five countries by Mokuwa et al. [29]. That some *glaberrima* varieties from Senegal and Guinea Bissau sit with those from Ghana and Togo when combining PC1 and 2 (Figure 3) might imply a process of adaptation to agro-ecological conditions, such as amount of rainfall, since this is comparable in the two regions.

Table 3 shows that *glaberrimas* from the Lower Guinea Coast have longer and heavier seeds than those from the Upper Guinea Coast. The differences in seed and plant height-related traits might be ascribed to a process of adaptation to specific ecological and/or socio-cultural factors. Farmers on the Danyi Plateau in the Togo Hills stated that *glaberrimas* used to thrive well on relatively poor and acid soils, in which the availability of vital nutrients is restricted. The cultivation of rice under these acid conditions might have led to selection for shorter plants that produce heavier and longer grains. Roy et al. [34] showed that larger seeds germinate better and produce more vigorous seedlings than smaller seeds and are able to produce a deeper initial root system. Also farmers on the Danyi plateau indicated that larger seed is clearly preferred for culinary reasons (B. Teeken, unpublished data).

A few *glaberrima* varieties from Ghana, Sierra Leone, Guinea and Guinea Bissau separate (downwards) from the core *glaberrima* cluster (Figure 3). These varieties have more tillers and panicles but have narrower leaves and smaller seed width compared to the other *glaberrimas*. For these traits, these *glaberrima* varieties resemble the *indica* group.

Unlike the case for *O. glaberrima*, no separate clustering can be observed for *O. sativa* ssp. *indica* from the Upper and Lower Guinea Coast (Figures 3, 4, 5, 6, 7, and 8), nor are significant differences observed for the agronomic traits (Table 3). At molecular level, some *indicas* from Sierra Leone and the Maritime region of Guinea tend to cluster together. Likewise, the materials from Senegal and the Togo hills cluster. However, at the morphological level a different tendency can be observed. Within the *indica* group (Figure 3) those from Guinea are situated towards the right, and those from Senegal are situated in the upper part, of a cloud. The *indicas* from Togo, Ghana, Sierra Leone and Guinea Bissau sit together in the centre of the cloud. The *indicas* show similarity with the farmer hybrids, particularly the semi-droopy hybrids from Sierra Leone and Guinea, and the droopy hybrids from Guinea Bissau.

Within Figures 3, 4, and 5, the *indicas* from Guinea closely bunch together whereas those from Ghana and other countries are very scattered. One explanation is that the materials collected in Guinea represent a small range of *indica* varieties, whereas a wide range of *indica* varieties was collected from rather diverse ecologies (ranging from hydromorphic soils to pure upland ecologies) in Ghana. Only when combining PC 1 and 4 (Figure 6) and PC 2 and 4 (Figure 7) is the Guinea material pulled apart, reflecting diversity on seed width and length, but not on other traits. The Guinea materials do not differ from the other *indica* varieties from the upper Guinea Coast on agronomic traits, except slightly for leaf length (Table 4).

Our findings at morphological level suggest that farmers in Ghana, Guinea Bissau, Sierra Leone and Senegal tend to select *indica* varieties with a range of morphological features while farmers in Guinea have been selecting narrowly, favouring a particular group of *indicas*. In the rather difficult upland conditions of the Guinea case-study areas (adjacent to the Bena hills) only a limited range of *indica* varieties has proven to be locally well adapted.

As is the case with *O. glaberrima, O. sativa* ssp. *japonica* from the Togo hills tends to have heavier seeds than the *japonica* from the Upper Guinea Coast region, but unlike *glaberrima* the *japonica* from the Togo hills are taller plants (Table 3). Considering PC 1 and 2 (Figure 3) the genotypes from the Upper Guinea Coast (mostly from Sierra Leone and Guinea Bissau) are found throughout the whole of the *japonica* cluster, while genotypes from the Lower Guinea Coast (materials from Ghana and Togo) are only found in the lower part of the cluster. *Japonica* varieties situated in the upper part of the cluster have broader leaves, fewer panicles and tillers, broader seeds and darker leaves. Materials from Sierra Leone and Guinea Bissau showed equal (high) levels of variation for these traits (see also Table 4). The *japonicas* from Sierra Leone were only collected from the south of the country meaning that farmers in a specific area deal with a highly diverse set of *japonica*s. At molecular level the *japonicas* from Sierra Leone tend to cluster separately from those from the other countries. Such separation does not show clearly in Figures 3, 4, 5, 6, 7, and 8.

**Figure 9:**
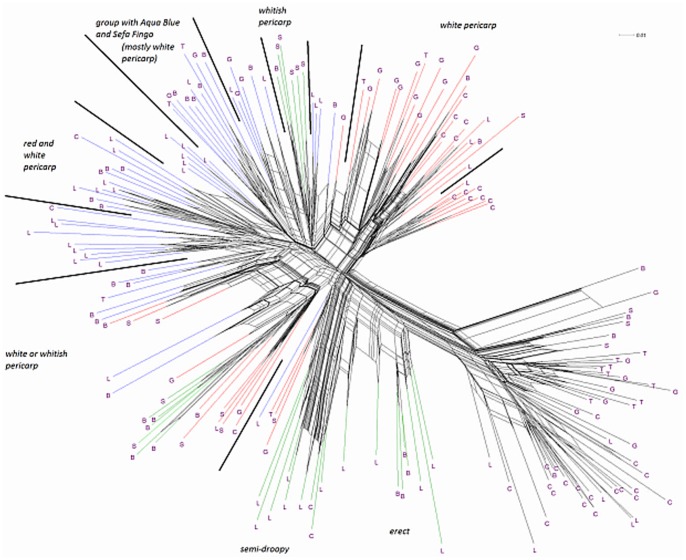
Phylogenetic relationships of 182 rice genotypes based on all morphological traits converted into dummy variables. Botanical groups are indicated by colours: Black  =  *O. glaberrima*, red  =  *O. sativa* ssp. *indica*, blue  =  *O. sativa* ssp. *japonica* and green  =  interspecific farmer varieties. Country of collection is indicated by letters: B  =  Guinea **B**issau, C  =  Guinea **C**onakry, G  =  **G**hana, L  =  Sierra **L**eone, S  =  **S**enegal, T  =  **T**ogo.

**Figure 10:**
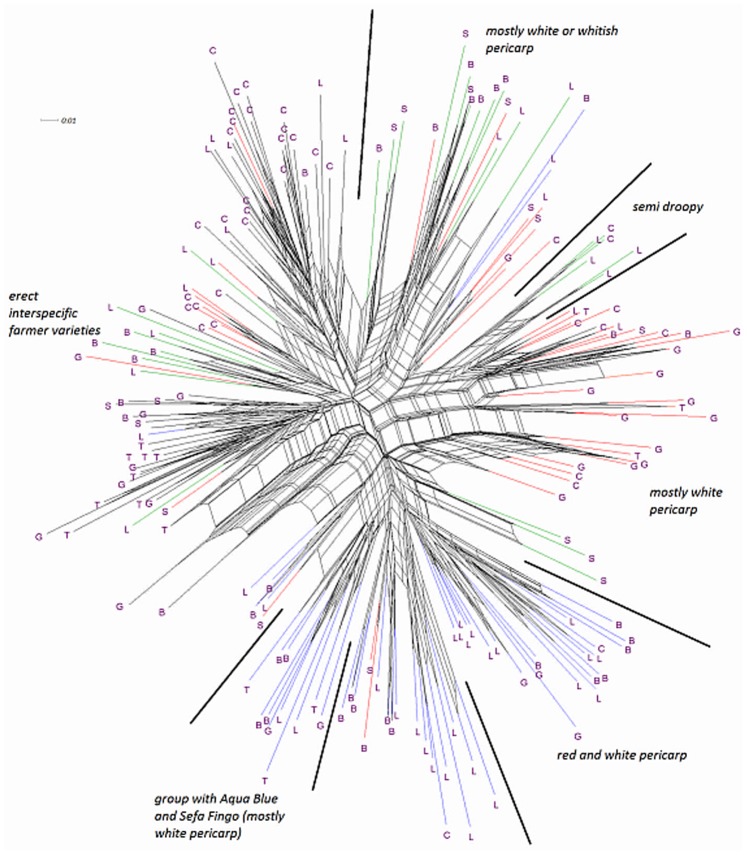
Phylogenetic relationships of 182 rice genotypes based on the agronomic traits converted into dummy variables. Botanical groups are indicated by colours: Black  =  *O. glaberrima*, red  =  *O. sativa* ssp. *indica*, blue  =  *O. sativa* ssp. *japonica* and green  =  interspecific farmer varieties. Country of collection is indicated by letters: B  =  Guinea **B**issau, C  =  Guinea **C**onakry, G  =  **G**hana, L  =  Sierra **L**eone, S  =  **S**enegal, T  =  **T**ogo.

Mokuwa et al. [10] found that a group of *japonicas* from Sierra Leone were more niche adapted, whereas a group of *japonicas* from Guinea Bissau showed wide adaptation. Both the Sierra Leone materials and most of the materials from Guinea Bissau used in the experiments by Mokuwa et al. [10] are among the genotypes sitting in the upper part of the *japonica* cluster (PC 1 and 2; Figure 3). At molecular level one Sierra Leone variety (Nduluwai) clusters with the Guinea Bissau varieties. In Figures 9 and 10 these varieties are found in different sub-groups, clustering in idiosyncratic ways. What this suggests is that farmers in both regions have selected morphologically similar materials responsive to different agro-ecological conditions. This might reflect histories of adaptation and introduction for these *japonicas* [29]. Evidence supporting a different process of introduction and adaptation is the similarity of the varieties Aqua Blue (‘blue water’) from Ghana and Sefa Fingo (meaning ‘black type’ in Mandinka) from Guinea Bissau at molecular and morphological levels, perhaps indicating common origins via Portuguese trading networks. It is thought that Portuguese traders brought *japonicas* from Indonesia to Guinea Bissau from where they spread to other West African countries [35]. To emphasise the distinctiveness of this case, both varieties have a distinct colouration during flowering and maturation not observed in other varieties.

Farmers from the Ghana side of the Togo hills have been selecting *japonicas* with relatively narrow leaves, high tillering and panicle production, slender and long grains similar to some *indicas*. (Figures 5 and 6). The long grain size could be explained by the large demand for long grained rice in the market.

In Figures 3, 4, 5, 6, and 7, farmer hybrids (Cluster 4) in general formed a large cloud suggesting they are diverse, confirming the molecular findings. Based on the panicle architecture (PAMA) most widely used to distinguish *O. glaberrima* from *O. sativa* varieties, the farmer hybrids were assigned to three sub groups: erect panicles, semi-droopy panicles and droopy panicles. In Figures 3, 4, 5, 6, and 7 the farmer hybrids with erect panicles did not clearly separate from the farmer hybrids with semi-droopy and droopy panicles, although they did in Figures 9 and 10. Table 4 shows statistically significant differences in seed weight, length and width between farmer hybrids with erect and semi-droopy panicles. For these two groups no clear difference was observed in the clustering based on molecular data.

Figure 4 (PC 1 and 3) shows that the farmer hybrids with erect panicles from Guinea Bissau cluster closely together, whereas those from Sierra Leone are more scattered. This agrees with the molecular analysis. Particularly, erect farmer hybrids from Northern Guinea Bissau sit together. These varieties were considered weeds by Mandinka farmers from northern Guinea Bissau; they referred to these interspecific varieties by names they also used for *glaberrima*. The one from Southern Guinea Bissau was brought from Guinea and sits somewhat separated. The scattering of the farmer hybrids with erect and semi-droopy panicles from Sierra Leone in Figure 4 points to active selection by farmers.

The erect farmer hybrids of Guinea Bissau and Sierra Leone are known to be four months in duration from germination to ripening. The semi-droopy farmer hybrids from Sierra Leone and Guinea are three months in duration. These semi-droopy farmer hybrids can be further divided into those with small and slender grains and those with short and bold grains. The latter are visible in Figure 6 down among the semi-droopy farmer hybrids from Sub-cluster 4-1. Farmers have been selecting ‘three month’ varieties as hunger breakers because they ripen about one and half or two month(s) before the major rice harvesting time. In this respect the three-month group of farmer hybrids has been replacing some of the short cycle *glaberrima* traditionally used as hunger breakers. Compared to the erect farmer hybrids these ‘three months’ interspecific farmer varieties vary more for husk colour and seed size (see also Table 4).

Compared to the limited diversity represented by the erect farmer hybrids from Guinea Bissau, the larger diversity in droopy farmer hybrids from Guinea Bissau and Senegal (Sub-cluster 4-2 in Table 4) agrees with interview data that farmers actively select for droopy farmer hybrids in these regions. The farmer hybrids with droopy panicles split into two groups largely reflecting their area of collection. Figure 3 (PC 1 and 2) shows that only farmer hybrids with droopy panicles from Senegal are found in the area where *indica* and *japonica* overlap. Interviews with farmers indicated that the farmer hybrids in Senegal have their origin in Guinea Bissau. The droopy varieties spread to Senegal particularly during the independence war in Guinea Bissau from 1963 to 1974. However, the farmer hybrids with droopy panicles collected in Guinea Bissau are situated in the lower part of the cloud of farmer hybrids. This suggests that over a period of approximately 40–50 years a selection process has taken place, and that farmers in the case study areas in Senegal and Guinea Bissau prefer farmer hybrids with area-specific morphological characteristics. Overall, morphological characterisation of farmer hybrids underlines a conclusion that Cluster 4-2 is highly variable and shares characteristics with *japonica* and *indica*. At the molecular level, however, the farmer hybrids are all closer to *indica* than to *japonica*.

### Geographical and Climatic Clustering

Figure 9 shows an unrooted tree based on 20 morphological traits, and Figure 10 based on agronomic traits only (see Table 5). The clustering in Figure 9 is largely according to botanical groups, with *glaberrima* and *japonica* making well-defined clusters and *indica* and the farmer hybrids consisting of several clusters. The *glaberrima* clearly forms three sub-clusters for Guinea and Sierra Leone, Ghana and Togo, and north Guinea Bissau and Senegal. For the other botanical groups some country based clustering patterns can also be observed, although less clearly. Some clusters contain material from several botanical groups. In the case of *japonica*, two clusters hold mostly material from Sierra Leone, and another cluster groups material from various countries. In the case of *indica* from the Upper Guinea Coast some clustering based on seed colour can be observed, but not for the Lower Guinea Coast. Some white seeded *indicas* cluster with light-coloured droopy farmer hybrids from Guinea Bissau, and some red seeded *indicas* cluster with red-coloured semi-droopy farmer hybrids from Sierra Leone and Guinea.

**Table 5:** The 20 traits measured on the rice genotypes in a field trial in Sierra Leone in 2008. Ratings were based on five at randomly chosen plants per plot.

Characteristic	Description and scale or unit	Type of determination	Stage of measurement
*Agronomic traits*			
Culm length	Average length, from ground level to the base of thepanicle, in cm	Numerical	Physiological maturity
Plant height	Average height, from soil surface up to the tip of the tallestpanicle, in cm	Numerical	Physiological maturity
Leaf length	Average length of peninsulate leaf (leaf below flag leaf),from collar to tip of leaf, in cm	Numerical	Physiological maturity
Leaf width	Average width of peninsulate leaf (leaf below flag leaf),widest portion of the leaf, in cm	Numerical	Physiological maturity
Panicle length	Average length, of main panicle, from panicle baseto tip, in cm,	Numerical	Physiological maturity
Panicle number	Average number of panicles per plant	Numerical	Physiological maturity
Number of tillers	Average number of tiller(s) per plant	Numerical	Physiological maturity
Rattoon potential	Assessed after harvests: 0 = None; 1 = Low; 3 = Medium;5 = Vigorous; 7 = Very vigorous	Scale.	After harvest
Grain length	Average length of grain length, from base of lowermoststerile lemma to tip of fertile lemma or palea, in mm.	Numerical	Post-harvest
Grain width	Average width, measured at the widest portion, in mm.	Numerical	Post-harvest
100-grain weight	Average weight of 100 filled seeds at 13% moisture content.	Numerical	Post-harvest
*Botanical traits*			
Leaf blade colour	0 = No green visible due to anthocyanin; 3 = Light green;5 = Medium green; 7 = Dark green	Visual assessment	Physiological maturity
Leaf blade pubescence	1 = Glabrous (smooth); 2 = Intermediate; 3 = Pubescent	Ocular inspection and thenfingertip rub to class hairiness	Physiological maturity
Ligule length	Average length, on peninsulate leaf of main stem, from thebase of the collar to the tip, in mm	Numerical	Physiological maturity
Ligule shape	0 = Absent; 1 = Truncate; 2 = Acute to acuminate; 3 = 2-cleft	Visual assessment	Physiological maturity
Panicle attitude of mainaxis (PAMA)	1 = Upright; 2 = Semi-upright; 3 = Slightly drooping;4 = Strongly drooping	Visual assessment of the mainaxis of the panicle	Physiological maturity
Panicle attitude ofprimary branches (PAB)	1 = Erect (compact panicle); 3 = Semi erect, semi-compactpanicle; 5 = Spreading (open panicle); 7 = Horizontal; 9 = Drooping	Visual assessment	Physiological maturity
Awn length	0 = None (awn less); 1 = Very short (<5 mm);3 = Short (∼8 mm); 5 = Intermediate (∼15 mm);7 = Long (∼30 mm); 9 = Very long (>40 mm)	The awn was measured frombase to the tip, then translatedin scales	Post-harvest
Husk (lemma and palea) colour	1 = White; 2 = Straw; 3 = Gold and gold furrows; 4 = Brown(tawny); 5 = Brown spots; 6 = Brown furrows; 7 = Purple;8 = Reddish to light purple; 9 = Purple spots; 10 = Purplefurrows; 11 = Black	Visual assessment	Post-harvest
Seed coat colour /pericarp colour	1 = White; 2 = Light brown; 3 = Speckled brown; 4 = Brown;5 = Red; 6 = Variable purple; 7 = Purple	Visual assessment	Post-harvest

The clustering in Figure 10 is complex, with material from the four botanical groups clustering in various ways. To some extent the patterns may reflect agro-ecological selection pressures. This is perhaps particularly true for the *glaberrimas,* where grouping reflects geographical factors. The *glaberrimas* from Senegal and northern Guinea Bissau, for instance, cluster more closely with the *glaberrimas* from Togo and Ghana, with both areas having similar amounts of rainfall. For the other botanical groups no such clear separation is apparent. Another apparent indicator of agro-ecological selection pressures is the extent to which material from various botanical groups from one country, or two neighbouring countries, clusters together. For example, the erect farmer hybrids, most coming from Sierra Leone, and the *indicas* from Guinea cluster closely with the *glaberrimas* from Guinea and Sierra Leone. However, clusters can also be found grouping material from all countries, as applies to subsets of *indicas* and *japonicas*. Also the droopy farmer hybrids from Senegal form several small independent clusters.

### Pericarp Colour as a Selection Factor

Seed colour (pericarp) is an important characteristic often mentioned by farmers [9].

**Figure 11:**
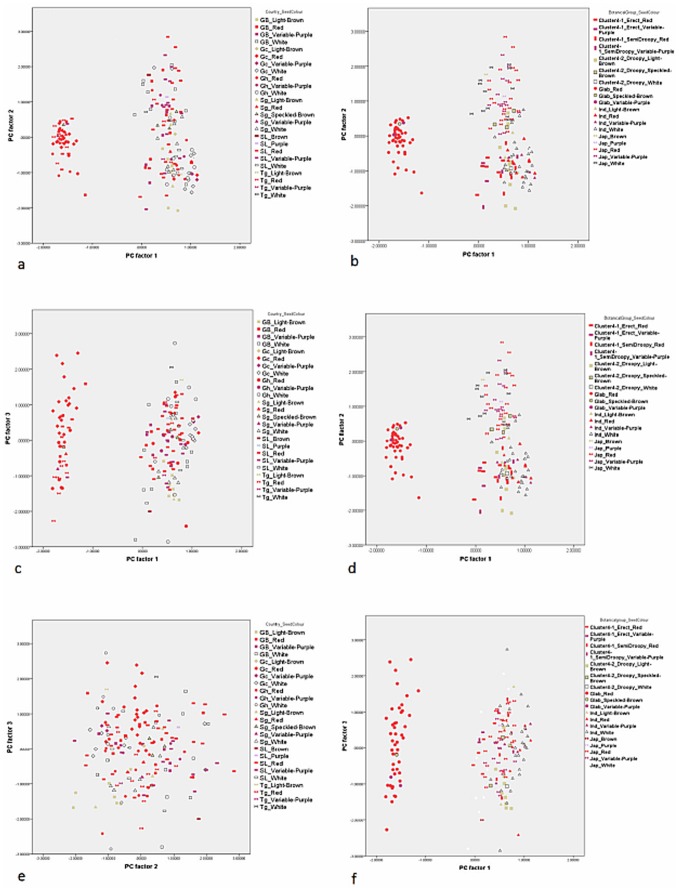
Graphically repartition of materials based on morphological data of PC 1&2, 1&3 and 2&3 marked according to country of collection and seed colour* (11a, 11c and 11e) as well as botanical group and seed colour (11b, 11d and 11f). Country of collection is indicated by letters: Gc  =  Guinea Conakry, GB  =  Guinea Bissau, Gh  =  Ghana, Sg  =  Senegal, SL  =  Sierra Leone, Tg  =  Togo. *According to the seed colour chart of the Bioversity rice descriptor version 2007 [49].

Depending on the farming system and social context, pericarp colour is a nutritional, gender, religious or cultural marker, and plays a role in the selection and acceptance of rice varieties [9]. Seed colour was not incorporated in the PCA because it could not be converted into a linear scale. Instead we labelled the materials of this study according to seed colour. Figure 11 shows the combination of factor PC 1 and 2, 1 and 3 and 2 and 3 marked according to seed colour, country of collection and botanical group. Only a few relationships between pericarp colour, molecular, and morphological data were found. The clearest relationship was among the farmer hybrids, where varieties with erect and semi-droopy panicles have a red pericarp, and those with a droopy panicle have a white or light brown pericarp. The varieties Pugulu ‘white’ and ‘red’ from Ghana were found in neighbouring clusters in Figure 9 (B. Teeken, unpublished). This is a case of farmers using the same name with the addition ‘white’ or ‘red’ for varieties that are genetically different.

Farmers from whom we collected material have no fixed ideas about the ‘correct’ morphological traits of rice varieties [36]. Rather than focusing on a particular ideotype they sustain what might be termed a broad flexset (combining a range of ideotypes depending on the conditions). Seemingly, this is a way to optimize benefits of cognitive flexibility, to be understood in relation to a long history of in situ domestication. Gross et al. [37] indicate that pericarp colour might be a phenomenon rather independent of trajectory of domestication. In Ghana preference for white and red varieties was modulated by other traits, such as robustness, yield and intended usage. In the Ghanaian Togo hills, as well as in Sierra Leone, rice with a red pericarp is considered ‘heavier’ in the stomach (i.e. it digests more slowly than white rice, a valuable characteristic where sustained hard work has to be attempted). To make a meal last longer, white rice is sometimes mixed with some red rice before eating and in some cases (e.g. in Sierra Leone) it is sometimes mixed before sowing to allow easy milling. In Mandinka-dominated areas of Upper West Africa, red rice is regarded as “outmoded” and white rice is now preferred. There is also high demand for white rice in urban areas where people, because of their different labour pattern, tend to prefer rice that is more easily prepared.

### Development of Genetic Diversity

Whereas the molecular data suggested that the *indica* group and farmer hybrids had greater genetic diversity than the *japonica* and *glaberrima* groups (see Table 6), Figures 3, 4, 5, 6, 7, and 8 suggest the differences in genetic diversity between the groups might be smaller than represented by the molecular analysis. Particularly for *japonica*, Figures 4, 5, 6, 7, and 8 and Figure 11 show a large dispersion, similar to *indica*, for all components. For *glaberrima* only Figures 4, 5, and 8 show a large dispersion. Calculations of genetic diversity based on morphological traits (Table 6) confirm that *glaberrima* have less diversity, but that *japonica* has a higher level of diversity (see also Tables 3 and 4).

**Table 6:** Level of genetic diversity of four botanical groups of rice in West Africa, calculated with Nei’s index (He) and the fraction of polymorphic markers (P-value), based on molecular markers and morphological traits converted into dummy variables.

Botanicalgroup	N	Molecular markers		Morphological traits	
		He	P-value	He	P-value
*O. glaberrima*	49	0.042	0.430	0.189	0.762
Lower Guinea Coast	17	0.027	0.181	0.131	0.438
Upper Guinea Coast	32	0.047	0.362	0.185	0.724
Guinea	19	0.052	0.305	0.154	0.610
Other	13	0.035	0.152	0.190	0.552
*O. sativa* ssp. *indica*	46	0.099	0.653	0.218	0.800
Lower Guinea Coast	17	0.102	0.410	0.195	0.619
Upper Guinea Coast	29	0.070	0.429	0.208	0.733
Guinea	13	0.055	0.276	0.173	0.581
Other	16	0.072	0.333	0.208	0.590
*O. sativa* ssp. *japonica*	56	0.054	0.481	0.238	0.819
Lower Guinea Coast	8	0.033	0.143	0.173	0.476
Upper Guinea Coast	48	0.053	0.400	0.240	0.810
Guinea Bissau	18	0.035	0.200	0.236	0.676
Sierra Leone	28	0.056	0.371	0.225	0.705
Cluster 4	31	0.102	0.444	0.257	0.752
Cluster 4-1	18	0.060	0.274	0.223	0.629
Cluster 4-1erect	8	0.038	0.143	0.149	0.400
Cluster 4-1semi-droopy	10	0.065	0.219	0.169	0.495
Cluster 4-2	13	0.078	0.281	0.204	0.581

There seems to exist a relationship between the level of farmer selection and seed exchange and the level of diversity in botanical groups. Farmer accounts of the introduction or in situ development of new varieties related mainly to farmer hybrids, *indica* and to a lesser extent *japonica*. No such account related to *glaberrima*. In recent years farmers in Ghana have developed an idea that the morphology of *glaberrima* is fixed, and Mandinka people in Senegal and northern Guinea Bissau consider *glaberrima* to be a rice belonging to history. Only in a few areas (e.g. southern Guinea Bissau) are farmers actively re-introducing varieties of *glaberrima* [9]. This suggests there is little current active farmer variety development for *glaberrima*. By contrast, accounts concerning introductions and further development of farmer hybrids and/or *indica* are especially numerous in all countries where the research took place ([6], [9], [36]; A. Mokuwa, unpublished data).

### Country-specific Findings

#### Sierra Leone

In Sierra Leone *japonica* varieties are extensively cultivated only in the southern half of the country while almost all upland varieties in the north are mainly farmer hybrids and *indica,* with a few *glaberrima*. This suggests that diversity of climate and agro-ecological conditions (upland and hydromorphic ecologies) is the main driver for selection of botanical groups [38]. The *japonica* in southern Sierra Leone and the farmer hybrids in northern Sierra Leone show considerable variation, suggesting that active cultivation plays a role in maintaining and developing genetic diversity. Close to the border with Guinea more extensive cultivation of *glaberrima* occurs with varieties that resemble varieties from Guinea, an area where *glaberrima* is still widely cultivated [7], [9]. An ethnic factor plays a part - the *glaberrima* were collected mainly among the Susu people who live on both sides of the border, linked by strong family ties and seed networking relationships.

#### Guinea

It is important to mention that in the Guinea case study area almost no *japonica* varieties are cultivated. In these conditions (soils of low pH) farmers mainly cultivate *glaberrima* and *indica* varieties. Among rice scientists it is thought that West African upland varieties are generally *japonica* rather than *indica* [35], [39], [40]. As a result, and in contrast to *japonica*, *indica* cultivars have yet to be fully evaluated regarding their adaptation to upland conditions in West Africa ([41]; [42], cited in [43]).

The *indica* varieties collected in Guinea showed less diversity compared to varieties collected from the other study countries. This limited diversity partly relates to a fieldwork circumstance - varieties were collected from an ethnically homogenous group (Susu) growing essentially the same set of varieties along a 120 km transect from the Sierra-Leone borders (Bassia) to Kindia. These Guinean *indicas* are morphologically strongly differentiated from both *glaberrima* as well as from *japonica*. This is despite the fact that in Guinea *indica* and *glaberrima* are cultivated in the same upland conditions. For Susu farmers, selecting morphologically distinct genotypes helps avoid variety mixtures in the field. This part of Guinea (the Benna region in particular) was historically involved in an international rice trade to Freetown when local slave-manned plantations replaced the Atlantic slave trade. The Freetown rice trade demanded white rice [29]. Keeping field homogeneity (a relic of this long-dormant trade) is a cultural and managerial value lingering for nearly two centuries in some of these Susu farming communities [7]. That *indicas* with white and red seed colour cluster differently helps to confirm the significance of these socio-economic and cultural selection preferences in influencing genetic make-up of rice.

That the *glaberrima* from Guinea also show much diversity, points to active selection of African rice in this region. Mouser et al. [29] suggest that this may be linked to the food security needs of newly founded maroon communities of self-emancipated slaves fleeing Susu rice plantations.

#### Senegal

Senegalese *indica* (and hybrid) varieties resemble *japonica* in having fewer tillers and panicles, broader leaves and seeds than the *indica* from the other countries in the study. The land farmers work in Senegal mostly comprises hydromorphic soils, but also some uplands. The low tiller number is probably related to the relative earliness of local varieties. All farmer hybrids collected in Senegal had a light coloured pericarp as farmers strongly selected against red pericarp. A few off-types (representing old varieties) rogued from collected samples clustered with red seeded varieties from Guinea and Sierra Leone. This can be taken as an indicator that localized farmer preferences changing over time can influence the genetic make-up represented by the varieties cultivated.

#### Guinea Bissau

The collected farmer hybrids cluster with *indicas* and have low similarity to *japonica* from Guinea Bissau. *Japonicas* were cultivated under upland conditions and the farmer hybrids tended to be more frequently cultivated in hydromorphic zones. However, respondents said that in the past, when more labour was available for bird scaring, farmer hybrids were also cultivated in the uplands. The droopy farmer hybrids are genetically different from the erect farmer hybrids. One reason is seed colour. Mandinka farmers are unlikely to select an off-type with an erect panicle to develop it into a variety since they associate erectness of the panicle with (undesirable) red pericarp. The *glaberrima* from northern Guinea Bissau clustered with those from Senegal while those from southern Guinea Bissau clustered with those from Guinea and Sierra Leone. Climatic conditions are clearly different between the case study villages in the north and south of the country, but account should also be taken of the fact that historical relationships differ. The north is oriented towards Senegal (Casamance) and the south is oriented towards Guinea.

#### Togo Hills (Ghana and Togo)

The relatively large diversity within the *indica*, *japonica* and *glaberrima* groups in the Togo Hills can be partly ascribed to the many different ecological niches found in a forested landscape that ranges from lowland to mountain where farmers take considerable advantage of intra-mountain basins for rice cultivation. These mountain basins offered the double advantage of more fertile soils and security. Rice diversity can thus be related to a history of refuge, displacement and enclaved social life in a region characterized by war and political instability. Seeking security, farmers strove to intensify farming on stony, acid and often sloping soils by emphasizing *O. glaberrima*, the only rice available at that time. More recent factors include the developments in farming over the past 50 years. Until the 1960s, the main rice producing ecology was upland, where mainly *glaberrima* varieties were cultivated. On the Ghana side of the range, farmers started to cultivate *indica* varieties in lowland areas from the 1960s onwards, while in the Togolese Togo Hills (mainly the Danyi Plateau) farmers continued - to this day- to cultivate solely *glaberrima* varieties, as no lowland varieties were available to them. Lowland rice farming in the foothills of the Ghanaian Togo Hills has meanwhile become a major activity. It has also resulted in farmers introducing some *indica* varieties to hydromorphic and upland conditions. The cultivation of *glaberrima* is still maintained today, especially for its role in customary rites, and as a significant part upland cultivation clearly continues to set criteria for the selection and development of *indica* rice varieties [9]. This also helps explain why no farmer hybrids are found in the Togo hills. Here *O. glaberrima* and *O. sativa* are separated in the landscape by altitude, not grown side by side, as they have been for centuries, in Upper West Africa. It should also be noted that local customary rites demand use of pure *glaberrima* for feeding and sacrifice. This acts as a disincentive to any farmer inclined to select off-types intermediate between *O. glaberrima* and *O. sativa*.

## Conclusions and Implications

### Main Conclusions

This paper has combined morphological and molecular data with socio-economic and cultural information to provide a better understanding of how cultivation practices combine with environmental pressures to shape rice diversity in six case study areas in coastal West Africa. Examples have been provided of how, per botanical group and case study area, these integrated data offer novel insights into the potential of neglected crop resources. The paper points both to the complexity of farmer rice genetic diversity management and to the significance of farmer innovation.

For *O. glaberrima* the molecular and morphological data largely agree with each other (see Figure 1). The morphological data showed clear differences in morphological features between *glaberrima* varieties from the Togo hills and the Upper Guinea Coast region, and between Guinea and the other countries of the Upper Guinea Coast. A relationship between genetic diversity and agro-ecology emerges. Farmers did not exchange *glaberrima* varieties over large distances, and we did not receive information about the development of new *glaberrima* varieties (the Guinea case excluded). What seems now to be true is that ethnic groups either stress the true-to-type maintenance of specific varieties or have abandoned the cultivation of *glaberrima* altogether.

For the other botanical groups a different picture emerges. Molecular and morphological data do not always agree. Particularly for the *japonica* group more diversity was observed at the morphological than at the molecular level. This could be caused by the possibility that the molecular markers used were more informative on other botanical groupings than on the *japonicas*. Taken in conjunction with the findings on differences in adaptation within *japonica* reported by Mokuwa et al. [10] the question arises about whether the *japonica* harbour more genetic diversity than observed. At the morphological level the three non-*glaberrima* botanical groups did not group geographically (by country, or groups of neighbouring countries). Particularly for *indicas* and the farmer hybrids, much evidence of recently introduced or newly developed varieties was recorded. Particularly with the farmer hybrids, seed colour has a clear relationship with the genetic make-up of rice varieties. Such a relationship is non-existent (or less clear) for the *japonica* group.

Apart from *glaberrima*, farmers seem ready to cultivate any variety of the other three botanical groups that meets a certain minimum set of criteria, such as plant height, time of ripening, seed colour and digestibility. Even these criteria are used flexibly, depending on the other advantages a variety may possess. For example, in general farmers prefer tall varieties. In Ghana this is because a long stem is considered easier for threshing. In southern Sierra Leone farmers mostly harvest by panicle and seek to avoid too much stooping and an aching back. Short plants, however, may not always be selected against if they have compensating advantages such as earliness [36].

For *glaberrima*, socio-cultural selection pressures seem to reduce diversity, particularly at a more local scale, while for the other botanical groups they seem to have an enhancing effect on genetic diversity. However, *glaberrima* still plays an important role in determining the selection criteria of farmers and shaping variety development pathways. For instance, farmers in northern Sierra Leone select farmer hybrids with erect panicles. This implies these farmer hybrids are selected according to standards established for *glaberrima* cultivation. Most *japonicas* in southern Sierra Leone have a red pericarp. This results from historically-specific socio-cultural selection pressures [29]. The farmer hybrids from Senegal and Guinea Bissau show much overlap with, respectively, the *japonicas* and the *indicas* in the PCA analysis. This is apparently related in part to shared agro-ecological conditions. The droopy panicle and light seed colour of farmer hybrids in this region also reflect a history of *O. sativa* cultivation. In sum, at a regional level, farmer hybrids combine (advantageous) traits from different botanical groups by embodying responses to different local cultural and ecological considerations.

Because farmers in West Africa embark on risk spreading practices - e.g. growing varieties mixed-in with other varieties and assigning sections of their fields to different varieties [44] – ‘in-situ’ experimentation and on-farm hybridization is facilitated. In Sierra Leone farmer hybrids are generally popular; they are said to perform well under low field management and when consumed enable farmers to sustain longer hours of work without hunger, and are thus similar to *glaberrima*. In Senegal, the farmer hybrids also perform well under low field management, but do not have a red pericarp and in that respect are regarded as being similar to the *O. sativa* varieties commonly planted. The farmer hybrids are a welcome enrichment of local planting resources since they are genetically rich and diverse and can be considered products of long trajectories of interaction between botanical groups, ecological, socio-cultural and economic factors.

### Wider Societal Context and Implications

The present paper belongs to a group of three that report an interconnected set of findings: we first described the emergence of a new rice type of interspecific hybrid origin in West African farmers’ fields [23], then we analysed robustness and strategies of physiological adaptation within a large set of farmer varieties of African rice and Asian rice across West Africa [10] and third, this paper has compared morphological and molecular data with information on socio-economic seed selection factors, in order better to show how farmer practices and culture combine with environmental selection pressures to shape diversity in rice across coastal West Africa.

All three papers provide evidence that West African small-scale food-crop farmers conserve and develop valuable rice varieties, despite limitations of poverty, isolation, and formal education. A major implication of this result is that farmer practices and culture strengthen the conservation and development of genetic diversity. Modern varieties of many crops have little or no genetic disparity within cultivars. It has been estimated that as little as 20% [45] of the total diversity contained within the wild ancestors of rice, cassava, and soybeans is maintained through breeding of ‘modern elite’ varieties [46]–[48]. Our work shows, by contrast, that farmer innovation helps to protect this diversity and keep it ‘in play’ for future adaptation. Sustaining crop genetic diversity in situ is an especially important topic in an era of rapid climatic change. Our results, therefore, support calls for the protection and valorisation of farmer crop innovation processes, as a basis for addressing issues of rural food security in Africa.

## Materials and Methods

### Ethics Statement

We confirm that no specific permit was required for using the location where the field trial was conducted. The location was not protected in any way. The field study never involved endangered or protected species. Approval for the collection of socio-economic data using in-depth interviews and questionnaires was obtained from the Social Sciences Ethical Committee (SSEC) of Wageningen University. The research was carried out by researchers living in the country for at least several years and approved by village elders and farmer communities. Individual participants provided their verbal informed consent to participate in the interviews as part of the interview protocol. Written consent was not possible as most of the interviewees were illiterate. The SSEC approved this consent procedure. We thank the village elders, farmers and the land holding family at Fala Junction Kowa Chiefdom, Sierra Leone.

### Variety Collection and Molecular Analysis

**Figure 12:**
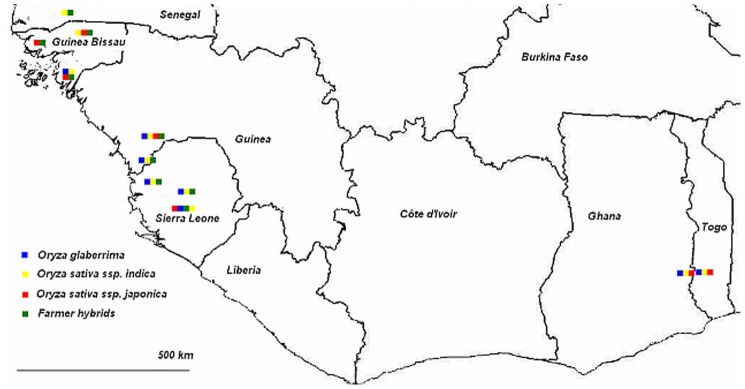
Case study areas are indicated by colours representing the most commonly cultivated botanical groups in those areas.

Variety collection was carried out from June to December 2007 in seven countries of Coastal West Africa: The Gambia, Ghana, Guinea, Guinea Bissau, Senegal, Sierra Leone and Togo (Figure 12). The purpose was to collect varieties of *O*. *glaberrima* and *O*. *sativa* cultivated by farmers in regions where *O. glaberrima* was known to be cultivated. In each country varieties were collected in a number of case study villages. In exceptional cases, varieties in other villages were collected if they had a clear relationship to the main case study villages, if there was an important ‘story’ related to them, or if they were morphologically intermediate between *O. sativa* and *O. glaberrima*. At harvest time a total of 231 accessions were collected. In February and March 2008 these accessions were analyzed molecularly using AFLP markers. In the research by Nuijten et al. [23] these data were then added to the 84 accessions analyzed by Nuijten and Van Treuren [30]. With the software package ‘Structure’ (version 2.2), materials with a probability equal to or higher than 91% were assigned to four clusters (*glaberrima*, *indica*, *japonica* and farmer hybrids (see Table 7). Materials assigned with a value lower than p =  0.91 were considered outliers. Farmer hybrids are farmer varieties of interspecific origin [23].

**Table 7:** Number of materials used in the molecular and morphological analysis according to their botanical group and their areas of collection.

Botanical group	Senegal (Casamance)	Guinea Bissau	Guinea (Kindiaand Forecariah)	Sierra Leone (Central- N/West)	Ghana (Togo Hills,Volta region)	Togo (Togo Hills,Danyi plateau)	Total
*O. glaberrima*	3	4	19	6	8	9	49
*O. sativa* ssp. *indica*	7	4	13	5	15	2	46
*O. sativa* ssp. *japonica*	0	18	2	28	5	3	56
Farmer hybrids*	7	10	2	12	0	0	31
Total	17	36	36	51	28	14	182

Interspecific farmer varieties with a combined background of *O. glaberrima* and *O. sativa.*

### Choice and Types of Farmer Varieties

In this paper we consider only the materials that were assigned with a probability equal to or larger than 91% to the botanical groups *O. glaberrima*, *O. sativa* ssp. *japonica*, *O. sativa* ssp. *indica* and the farmer interspecific hybrids (Cluster 4). The focus of this study was on upland varieties. Apart from pure upland varieties also varieties from the upper part of the lowland-upland continuum were included. Typical lowland varieties were left out.

In addition, the number of materials collected from The Gambia in 2007 was too limited for a meaningful comparison and were left out. Because for some materials not enough seeds were available for the morphological analysis, we worked with a total of 182 varieties.

### Trial set-up

Field evaluations were carried out in Sierra Leone from June to December 2008. The trial was set up under upland rain-fed conditions at Fala Junction, Kowa Chiefdom (8.14917 N, 11.90806 E, 58 m asl), in Moyamba District. The period of field evaluation corresponded to the cropping season. The average annual rainfall is between 2100–3000 mm and the rainy season lasts for 6 to 7 months. The selected site was flat. The soil was cleared and deeply plowed after 24 years of bush fallow. The soil was silt loam (Mende: *tumui*).

The seeds of each accession were sown in a randomized block design. Each plot was 1.5 m × 2.1 m and contained 70 pockets, spaced 30 cm between rows and 15 cm within rows. Three grains were sown in each pocket and pockets were thinned to one plant within four weeks after sowing. Sowing date was determined by following the farmer practices in the region. Excellent germination and growth were observed with low to moderate pest (rodent, termites, cut worms, stem borers) incidences, mostly with *O. sativa* ssp. *japonica* varieties. Traditional fencing and mesh wire were used to prevent damage by rodents. No fertilizer was applied.

### Measurements

A total of 20 traits were measured (Table 5). Most traits were measured in all four replications, except a few qualitative traits which were measured only on the first replication, as these traits were not influenced by microenvironment. Measurements were done on five plants chosen randomly in each plot excluding the border rows. The accessions were characterized according to the descriptor list by Bioversity International (2007) [49] with the exception of rattooning potential.

### Socio-economic Data Collection

Besides the collection of farmer accessions, socio-economic data were collected on all 182 varieties using in-depth interviews and questionnaires which mainly covered (i) household data, (ii) number of varieties grown, (iii) ecology of cultivation, (iv) the area under cultivation, (v) farmer reasons for growing the variety, (vi) seed source, (vii) on-farm seed management practices from harvest to sowing and farmer knowledge related to variety use.

### Data Analysis

Principal component analysis (PCA) was used to describe the morphological data measured through a reduced number of variables shown in biplot as vectors. The genetic implications can be assessed from the eigenvalues ascribed to the different traits [50]. The values of the principal components per genotype correspond to a combination of traits explaining the variability. The closer the distance between genotypes in the biplots with the different principal components the closer the genotypes are related with respect to the traits represented by the principal components. PCA was conducted using SPSS/ PASW Statistics 18.

The morphological data were also analysed with the software Splitstree [51]. The measured data were translated into dummy variables. For the data with ordinal scales: for each value a column was created. For the numerical data, the number of categories was determined based on the difference between the maximum and minimum value divided by the standard deviation. The width of a category was determined by dividing the range by the number of categories multiplied with the factor 1.5. These data and the molecular data were analysed with the software Splitstree using the same method followed by Nuijten et al. [23].
